# CROSS-SECTIONAL AND LONGITUDINAL ASSOCIATIONS BETWEEN SLEEP DISTURBANCES, DEPRESSIVE AND ANXIETY SYMPTOMS

**DOI:** 10.1093/geroni/igad104.2432

**Published:** 2023-12-21

**Authors:** Fanghong Dong, Nancy Hodgson

**Affiliations:** University of Pennsylvania, Philadelphia, Pennsylvania, United States; University of Pennsylvania School of Nursing, Philadelphia, Pennsylvania, United States

## Abstract

**Introduction:**

Older adults often experience elevated sleep disturbances, depressive and anxiety symptoms. However, the role of sleep disturbances in explaining individual variability in depressive and anxiety symptoms among older adults is poorly understood.

**Methods:**

The sample was derived from the National Social Life, Health, and Aging Project, a nationally representative longitudinal study among American older adults. MCI was defined as Montreal Cognitive Assessment scored less than 23. Subjective insomnia symptoms and objective sleep measures (total sleep time, wake after sleep onset, percentage sleep) obtained from actigraphy were used. Validated measures on depressive and anxiety symptoms were collected both at round 2(N=645) and round 3(N=456). Multiple regressions were conducted to establish cross-sectional and longitudinal associations between sleep disturbances, depressive and anxiety symptoms. Results Cross-sectionally, compared to cognitively intact older adults, severe insomnia symptoms were associated with poorer depressive symptoms(B=0.40, p< 0.01) in older adults with MCI. Severe insomnia symptoms were associated with poorer anxiety symptoms(B=0.13, p< 0.01) in older adults, and no interaction effects were found by MCI groups. Longitudinally, insomnia symptoms at round 2 were associated with poorer depressive symptoms and poorer anxiety symptoms in older adults at round 3, but no interaction effects were found by MCI groups. No significant relationships were found between objective sleep disturbances and depressive/anxiety symptoms both cross-sectionally and longitudinally.

**Conclusions:**

The findings provide further insight into insomnia symptoms that may be associated with increased risks for developmental depressive and anxiety symptoms. These data suggest that targeting insomnia treatment may confer long-term mental health benefits.

